# Multimodal contrastive learning for non-invasive chondroid bone tumor classification and grading using radiographs

**DOI:** 10.1186/s12880-026-02239-w

**Published:** 2026-02-20

**Authors:** Peiying Hua, Jessica M. Sin, Eric R. Henderson, Jason Ha, Darcy A. Kerr, Saeed Hassanpour

**Affiliations:** 1 Department of Biomedical Data Science, Geisel School of Medicine at Dartmouth, Hanover, NH 03755 USA; 2 Department of Radiology, Dartmouth-Hitchcock Medical Center, Lebanon, NH 03756 USA; 3 Department of Orthopaedics, Dartmouth-Hitchcock Medical Center, Lebanon, NH 03756 USA; 4 Department of Pathology and Laboratory Medicine, Dartmouth-Hitchcock Medical Center, Lebanon, NH 03756 USA; 5 Department of Epidemiology, Geisel School of Medicine at Dartmouth, Hanover, NH 03755 USA; 6 Department of Computer Science, Dartmouth College, Hanover, NH 03755 USA

**Keywords:** Chondroid bone tumor, Computer-aided diagnosis, Contrastive learning, Medical imaging, Multimodal AI

## Abstract

**Objective:**

Diagnosing and grading chondroid bone tumors with radiography is difficult due to overlapping features with benign conditions such as avascular necrosis and fibrous dysplasia. Histology remains the gold standard, but it is invasive, costly, and not always accessible. This pilot study introduces a contrastive learning framework to address two challenges: (1) developing an AI system for accurate tumor classification and grading using radiographs alone, and (2) creating an enhanced multimodal pipeline when histology is available.

**Materials and methods:**

We retrospectively analyzed radiographs from 188 patients and histology images from 63 patients at a tertiary academic medical center in the United States. The stepwise framework included: (1) unimodal classification, (2) contrastive learning to align cross-modal embeddings, and (3) classification using enhanced representations. Models were trained with 5-fold cross-validation and evaluated using AUC, accuracy, sensitivity, and specificity.

**Results:**

Our framework demonstrates promising performance across all tasks. The radiograph-based model achieved an AUC of 0.91 (95%CI: 0.82–1.00) in distinguishing tumors from avascular necrosis and fibrous dysplasia. For grading, contrastive learning improved radiograph-only performance from AUC 0.86 to 0.95 (95%CI: 0.85–1.00). The histology-only model improved from AUC 0.73 to 0.83 with contrastive enhancement. Multimodal integration achieved perfect discrimination (AUC = 1.00) on the available subset.

**Conclusion:**

This study establishes proof-of-concept that contrastive learning can effectively bridge radiographic and histological representations for tumor assessment. The framework offers clinical potential by enabling non-invasive classification in resource-limited settings while allowing multimodal enhancement. These results warrant validation in larger, multi-institutional cohorts.

**Supplementary Information:**

The online version contains supplementary material available at 10.1186/s12880-026-02239-w.

## Introduction

Chondroid bone tumors are neoplasms characterized by tumor cells differentiating toward cartilage (chondrocytes) with a cartilaginous matrix. They include benign forms like enchondromas and osteochondromas, and malignant forms like conventional chondrosarcoma, which are among the most common primary bone malignancies [1, 2]. Though rare, they are clinically important because they can weaken bone, impair mobility, and metastasize. Each type of chondroid bone tumors has distinct biological features and treatment needs, making accurate diagnosis and classification essential [3, 4]. 

Despite advances in imaging, distinguishing chondroid tumors from mimics such as avascular necrosis (AVN) and fibrous dysplasia remains difficult, particularly on radiographs. These conditions share overlapping features including irregular margins, calcifications, and variable signal intensities [5, 6]. Tumors may resemble AVN, while bone infarcts can mimic ring-and-arc calcifications typical of cartilaginous lesions. Fibrous dysplasia adds complexity with variable appearances [7, 8]. Differentiating low- from high-grade tumors (Soft Tissue and Bone Tumours.

WHO Classification of Tumours, 5th Edition [9]) is also difficult radiographically, as some benign lesions mimic aggressive disease [4, 10]. 

The clinical management of chondroid bone tumors, AVN, and fibrous dysplasia varies considerably. Benign tumors often need no treatment, low-grade chondrosarcomas usually require intralesional excision, and high-grade forms require wide resection [1, 4]. AVN treatment depends on stage, ranging from conservative care to core decompression or joint replacement [11, 12], while fibrous dysplasia is generally managed conservatively [8, 12, 13]. Misdiagnosis or delays may result in poor outcomes, underscoring the need for accurate, timely differentiation.

Biopsy followed by histological examination remains the gold standard for diagnosing chondroid bone tumors [14]. However, it is invasive, costly, and dependent on expert interpretation. Poorly performed biopsies can compromise margins or increase tumor seeding [15, 16]. These issues are especially critical in low-resource settings. Even with biopsy, grading is difficult due to heterogeneity, sampling bias, and variability among pathologists [17, 18]. 

Artificial intelligence (AI) and radiomics offer promising avenues for improving diagnostic and grading accuracy [19, 20]. Deep learning has shown success in detection, segmentation, grading, and prognosis using radiograph, CT, MRI, and PET [19–24]. Yet most models rely on histology or costly modalities, limiting scalability. AI systems effective with radiographs while incorporating histology when available would improve cost-effectiveness and accessibility [25, 26]. 

To address these challenges, we developed a contrastive learning framework that utilizes contrastive learning to enhance feature representation through cross-modality feature alignment. This study had two aims: (1) building a contrastive learning–assisted AI framework for classification and grading using radiographs alone, offering a non-invasive adjunct diagnostic and grading tool; and (2) constructing a multimodal pipeline to enhance grading when histology is available. By aligning histology and radiography in a shared feature space, the framework enables accurate radiograph-only classification while supporting multimodal enhancement.

## Materials and methods

### Datasets

This retrospective pilot study collected conventional radiological and histopathological images from patients with chondroid bone tumors, fibrous dysplasia, or avascular necrosis (AVN) at Dartmouth-Hitchcock Medical Center, a tertiary academic medical center in the United States. The final dataset included radiographs from 188 patients and histology slides from 63 patients. Inclusion required confirmed diagnoses and pre-treatment radiography or histology. Chondroid bone tumor cases were included if pathologically confirmed or if radiographic findings remained stable ≥ 2 years. Because biopsy was not routinely performed for AVN, fibrous dysplasia, or confirmed enchondromas, only 50 patients had both modalities.

The dataset encompassed various chondroid tumor types and grades. Among radiographic cases, 81 (43%) had chondroid tumors, 83 (44%) AVN, and 24 (13%) fibrous dysplasia. Of chondroid cases, 13 (16%) were high-grade malignancies requiring wide resection; 68 (84%) were low-grade (56 benign, 12 locally aggressive). AVN and fibrous dysplasia served as controls to train differentiation from radiographic mimics. Table 1 summarizes the dataset. Cohorts were split into training/validation/test at 64%:16%:20% using diagnosis-stratified sampling.

Ethical approval was obtained from the host institution’s Institutional Review Board. Given the retrospective, de-identified nature of the data, informed consent was waived.

**Table 1:** Patient distribution by diagnosis and imaging modality

Diagnosis	Radiographs (*n* = 188)	Histology slides* (*n* = 63)
High-grade chondroid bone tumor†	13	15
Low-grade chondroid bone tumor‡	68	48
Avascular necrosis or fibrous dysplasia	107	—

*Histology images were obtained only for chondroid bone tumor patients

†High-grade malignant chondroid bone tumors requiring wide surgical resection

‡Low-grade chondroid bone tumors including benign, locally aggressive, or low-grade malignant lesions

### Data preprocessing

All imaging underwent modality-specific preprocessing. Radiographs were converted from DICOM to PNG (Weasis Viewer). A fellowship-trained musculoskeletal radiologist (> 10 years experience) selected the most informative view and annotated regions of interest (ROIs) with bounding boxes using VIA annotator. ROIs were extracted, resized to a fixed input size, and normalized to standardize pixel intensity, ensuring uniform dimensions and resolution across samples. For histology, whole-slide images were stain-normalized. Tissue masks identified clinically meaningful areas and excluded background/artifacts. Slides were divided into non-overlapping 256 × 256-pixel patches; patches without sufficient tissue were discarded. This patch-based approach enabled efficient processing and fine-grained analysis.

### Single modality models

#### Radiograph-based model

For the radiograph modality, we used a transfer learning approach using the InceptionV3 architecture pretrained on the ImageNet dataset [27]. The original classification layers were removed and replaced with a task-specific head consisting of fully connected layers for diagnosis and grading. Given the limited dataset size and heterogeneity of bone tumor radiographs, the convolutional backbone of InceptionV3 was kept frozen throughout all training stages, including supervised learning and subsequent contrastive learning. This design choice preserved general-purpose visual representations learned from large-scale natural image data while reducing overfitting risk. Only the task-specific layers downstream of the backbone were optimized using radiograph data.

#### Histology-based model

Histology was processed with a ResNet-18 feature extractor followed by a transformer module to learn spatial relationships indicative of disease type and grade [28]. Whole-slide images were patched as above. Patch features from ResNet-18 were fed to a 12-block vision transformer (8 heads each), initialized with weights pretrained on The Cancer Genome Atlas histology dataset [29], then fine-tuned on chondroid bone tumor slides. Attention enabled learning of fine-grained cellular and large-scale architectural patterns. The model produced a 32-dimensional patient-level embedding for downstream contrastive learning and multimodal fusion.

To address class imbalance, we used focal loss [30] for all classification models except the contrastive step. Focal loss emphasizes hard, minority-class examples by down-weighting easy ones, thereby improving prediction accuracy for minority classes:

$$\:{\mathcal{L}}_{Focal}=\:-{\left(1-{P}_{t}\right)}^{\gamma\:}log\left({P}_{t}\right)$$

where $\:{P}_{t}$ is the predicted probability of the true class and γ(≥ 0) determines the extent to which hard examples are emphasized.

#### Contrastive learning encoders

To align representations from histology and radiographs, we used modality-specific multilayer perceptron (MLP) encoders to project backbone-extracted embeddings into a shared feature space. These MLP encoders serve as the primary trainable components during contrastive learning and are responsible for cross-modal feature alignment. Importantly, the convolutional radiograph backbone (InceptionV3) and histology feature extractor remained frozen during this stage.

Each encoder was followed by a nonlinear projection layer to improve feature compatibility before applying the contrastive objective. During downstream classification, the projection heads were discarded, and embeddings produced by the backbone followed by the trained MLP encoders (prior to projection) were used as input to classification layers. Throughout the manuscript, the term “enhanced embeddings” refers to these MLP-transformed representations, which encode cross-modal semantic information despite originating from single-modality features.

During contrastive learning, histology–radiograph pairs from the same patient served as positives, while mismatched pairs were negatives. The loss encouraged alignment of positive pairs and separation of negatives by maximizing cosine similarity for paired embeddings and minimizing it for unpaired ones, supporting robust cross-modal learning.

The contrastive objective combined InfoNCE loss with a focal loss component:

$$\:{\mathcal{L}}_{Contrastive}=\:\lambda\:*{\mathcal{L}}_{InfoNCE}+(1-\lambda\:)*{\mathcal{L}}_{Focal}$$

Where $\:{\mathcal{L}}_{InfoNCE}$ operates on paired projected embeddings to enforce cross-modal alignment, and $\:{\mathcal{L}}_{Focal}$ is applied to single-modality classification logits produced from the MLP-transformed embeddings to preserve class-discriminative structure during contrastive pretraining. The weighting parameter 𝜆∈[0,1] balances cross-modal alignment and supervised discrimination.

#### Single-modality disease classification with enhanced embeddings

A key objective of this study was to test whether accurate disease classification could be achieved using only radiographs, avoiding invasive and costly biopsy and histopathology. In this single-modality framework, the radiograph encoder extracted modality-specific embeddings, which were processed through the trained radiograph-specific MLP encoder. This produced enhanced representations optimized during contrastive learning.

These embeddings were then input into fully connected layers trained with focal loss to generate classification outcomes. The framework used only the backbone encoder and MLP encoder, excluding the projection layers from contrastive alignment. This allowed relevant diagnostic information to be extracted from radiographs alone, providing a potential non-invasive alternative to histology. The framework addresses the need for accurate tumor classification in settings where biopsy may be contraindicated, unavailable, or unnecessarily invasive.

#### Multimodal disease classification with enhanced embeddings

While radiographs offer a less invasive diagnostic option, distinguishing high- from low-grade chondroid bone tumors remains difficult, even with both radiological and histological information. To address this, we developed a multimodal classification framework integrating radiographic and histological data.

In this framework, modality-specific encoders extracted embeddings from each modality, which were processed through their respective MLP encoders to generate contrastive-aligned representations. Multimodal fusion was performed at the feature level by concatenating the MLP-transformed embeddings from each modality (i.e., mid-level feature fusion), and the resulting joint representation was passed to fully connected layers for final classification.

This multimodal approach leverages the complementary strengths of radiographs and histology to improve accuracy in scenarios where single-modality information is insufficient. By combining morphological features from histology with structural patterns from radiographs, the framework captures a more comprehensive diagnostic signature reflecting tumor biology and grade.

### Model training and evaluation

**Fig. 1:**
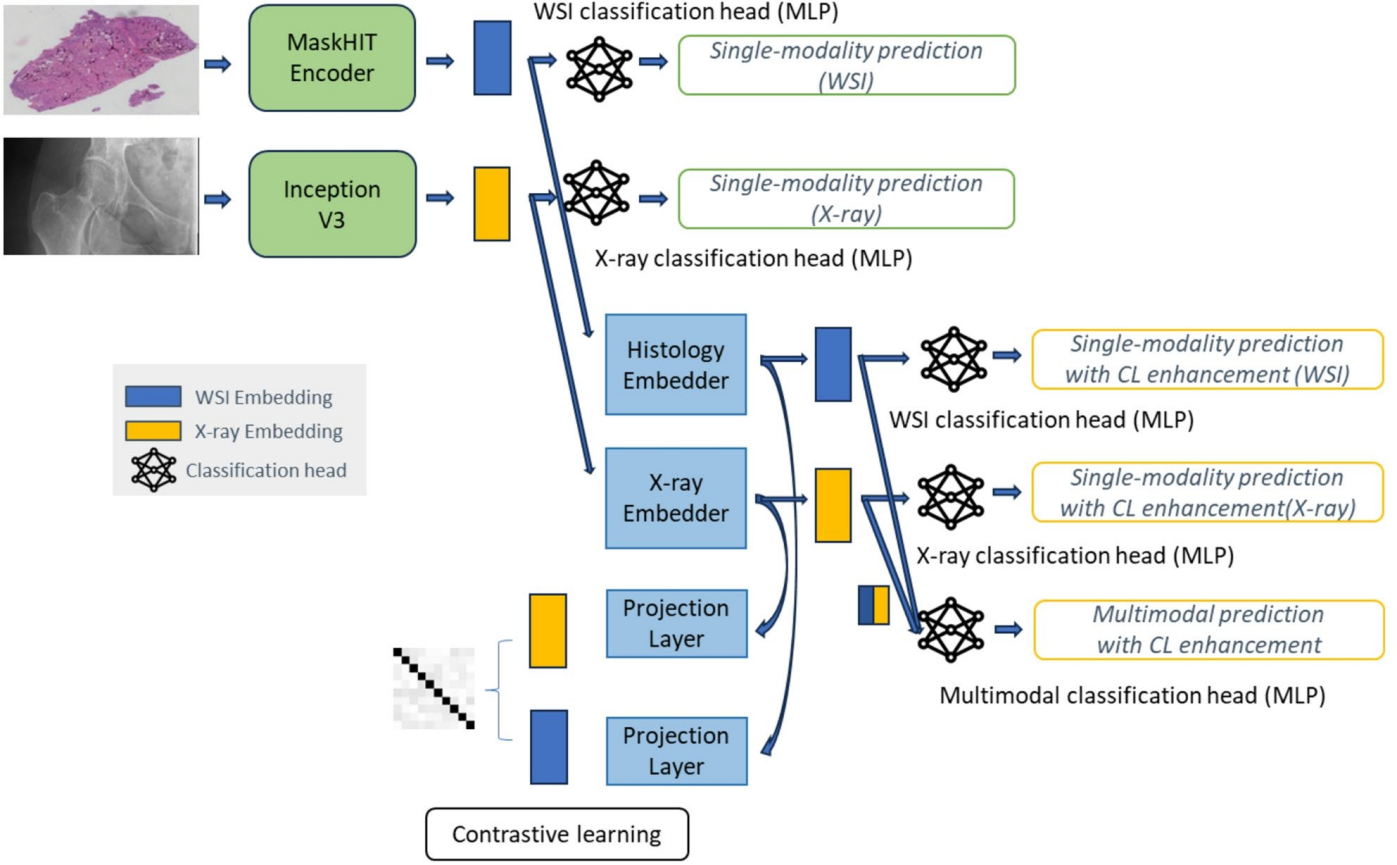
Overview of the multimodal AI pipeline for chondroid bone tumor classification and grading. Whole-slide histology images and radiographs are processed through modality-specific encoders (MaskHIT and InceptionV3) to generate initial embeddings for direct single-modality predictions. Enhanced embeddings are created through contrastive learning using MLP embedders and projection layers to achieve cross-modal alignment. The framework supports radiograph-only classification for non-invasive diagnosis, histology-only classification for tumor grading, or multimodal analysis combining (mid-level fusion of enhanced single-modality embeddings) both imaging modalities with contrastive learning enhancement

Model training and evaluation followed three steps (Fig. 1). Step 1: supervised training of modality-specific encoders with focal loss to learn diagnostic embeddings. Step 2: contrastive learning aligned radiograph and histology embeddings using modality-specific MLPs and projection heads, combining InfoNCE and focal losses to preserve discrimination strength while improving cross-modal consistency. Step 3: classification layers were trained on enhanced embeddings with focal loss to generate predictions.

Model training and hyperparameter tuning were conducted using 5-fold cross-validation, with batch size of 16, and early-stopping patience of 10 The final model ensembled the best-performing models from each fold and was evaluated on a leave-out testing set (80% training: 20% testing). All data splits were kept consistent in all steps to avoid information leakage. Dropout and L2 regularization were incorporated to mitigated overfitting. Performance was assessed by AUC, accuracy, sensitivity, and specificity. DeLong’s test compared results against chance, evaluated pre- vs. post-contrastive enhancement, and computed 95% confidence intervals, with Clopper-Pearson method used for computing 95% confidence intervals of non-AUC metrics.

### Ablation studies

To examine the impact of architecture and training strategies, we conducted ablation studies on radiograph- and histology-based models.

#### Radiograph model architecture comparison

We evaluated several CNN backbones—InceptionV3, EfficientNet (B1, B3, B5, B7), ResNet-18, and ResNet-50—for radiograph-based classification and grading. Smaller models (ResNet-18, EfficientNet-B1/B3) were fine-tuned with reduced learning rates on pretrained weights, while larger models (InceptionV3, ResNet-50, EfficientNet-B5/B7) had pretrained layers frozen to improve generalization with limited data. All models were trained with identical data splits for fair comparison. Performance was assessed primarily by AUC.

#### Histology model design choices

We compared two publicly available pipelines, Prov-GigaPath [31] and MaskHIT [28], both pretrained on large histopathology datasets. Each used transformer architectures with self-attention to emphasize diagnostically relevant regions without manual annotations. We also tested freezing versus fine-tuning pretrained encoders to assess the effect of adaptation on tumor grading performance.

## Results

### Model performance for bone tumor diagnosis and grading using radiographic images

#### Bone tumor diagnosis on radiographs without contrastive learning

An InceptionV3-based binary classifier was trained to distinguish tumors from two common mimics—fibrous dysplasia and AVN—using conventional radiographs as the sole input. On the test set, the model achieved an AUC of 0.91 (95% CI: 0.82–1.00), demonstrating promising performance. DeLong’s test confirmed accuracy above random chance (*p* < 0.001), validating overall diagnostic capability. Additional metrics were accuracy 0.87, sensitivity 0.82, and specificity 0.91. Complete evaluation results are summarized in Table 2.

**Table 2:** Performance metrics for radiograph-based models for chondroid bone tumor diagnosis and grading. All values represent point estimates with 95% confidence intervals in parentheses

Model	AUC	Accuracy	Sensitivity	Specificity	DeLong’s Test *p*-value
Diagnosis model	0.91 (0.82, 1.00)	0.87 (0.73, 0.96)	0.82 (0.57, 0.96)	0.91 (0.71, 0.99)	< 0.001†
Grading model without CL*	0.86 (0.62, 1.00)	0.88 (0.64, 0.99)	0.67 (0.09, 0.99)	0.93 (0.66, 1.00)	< 0.001†
Grading model with CL*	0.95 (0.85, 1.00)	0.88 (0.64, 0.99)	1.00 (0.29, 1.00)	0.86 (0.57, 0.98)	0.36 ǂ

*CL = contrastive learning

† Comparing against random chance

ǂ Comparing against grading model without contrastive learning

#### Bone tumor grading on radiographs

##### Model performance without contrastive learning

To complement our diagnostic model, we developed a treatment-oriented grading model to classify chondroid bone tumor grades (low-grade versus high-grade) based solely on radiographs. This approach targets the more granular clinical challenge of differentiating tumor grades without access to histological data. The grading model without contrastive learning achieved an AUC of 0.86 (95% CI: 0.62, 1.00), indicating promising performance for supporting treatment decisions in scenarios where biopsy or advanced imaging is unavailable or delayed. DeLong’s test confirmed statistically significant predictive capability for tumor grading (*p* < 0.001). The model demonstrated an overall accuracy of 0.88, specificity of 0.93, and sensitivity of 0.67. The relatively lower sensitivity likely reflects the limited proportion of high-grade cases in our dataset. Complete evaluation results are presented in Table 2.

##### Model performance with contrastive learning

To improve performance with added clinical context, we introduced a second training step using multimodal contrastive learning. This aligned embeddings across modalities, enhancing the radiograph encoder for tumor grading. The contrastive-enhanced model achieved a numerically improved AUC of 0.95 (95% CI: 0.85–1.00). This performance gain, with further validation, shows that contrastive learning can transfer discriminative features from histology to the radiograph domain without requiring histology images at inference time. The comparison of t-SNE plot visualization of embedding clustering before and after contrastive learning also shows a tighter clustering of patients deceased within 5 years after contrastive learning (Supplementary Fig. 1). DeLong’s test confirmed predictions were above chance (*p* < 0.001). The model achieved accuracy 0.88, specificity 0.86, and sensitivity 1.00 (Table 2). Compared to the radiograph-only model, sensitivity was numerically improved (1.00 vs. 0.67) while maintaining reliable specificity (0.86 vs. 0.93). With further validation on larger dataset, this gain can be clinically important, as missing high-grade disease could delay surgery and worsen outcomes. Notably, sensitivity estimates for high-grade tumors were derived from a very small number of cases per cross-validation fold, resulting in wide confidence intervals. Accordingly, the observed performance gains should be interpreted as directional and hypothesis-generating, consistent with the pilot nature of this study. DeLong’s test comparing models with and without contrastive learning did not show a significant difference (*p* = 0.36), likely due to limited sample size reducing statistical power despite observed AUC gains.

### Model performance for bone tumor grading using histology images

Even after obtaining histology slides through costly and invasive biopsy procedures, grading remains a complex and challenging task. In this section, we present the performance evaluation of our models for tumor grading using histology images alone and in combination with radiographic imaging within a multimodal framework.

#### Tumor grading performance on histology images without contrastive learning

We first trained a vision transformer model on whole-slide histology images for chondroid bone tumor grading. Designed to distinguish high- from low-grade tumors without manual annotation, the model achieved an AUC of 0.73 (95% CI: 0.39–1.00), with accuracy 0.69, specificity 0.80, and sensitivity 0.33 (Table 3), indicating moderate performance. However, DeLong’s test did not show significance above chance (*p* = 0.09). These results establish feasibility for automated histology-based grading, but the relatively low AUC and sensitivity suggest that additional contextual or complementary information is needed for clinically actionable performance.

**Table 3:** Performance metrics for histology-based models for chondroid bone tumor grading with optional radiographic integration. All values represent point estimates with 95% confidence intervals in parentheses

Model	AUC	Accuracy	Sensitivity	Specificity	DeLong’s Test *p*-value
Histology-only without CL*	0.73 (0.39, 1.00)	0.69 (0.39, 0.91)	0.33 (0.01, 0.91)	0.80 (0.44, 0.97)	**0.09†**
Histology-only with CL*	0.83 (0.60, 1.00)	0.77 (0.46, 0.95)	1.00 (0.29, 1.00)	0.70 (0.35, 0.93)	**0.002†**
Multimodal with CL*	1.00 (NA) ǂ	1.00 (0.72, 1.00)	1.00 (0.29, 1.00)	1.00 (0.63, 1.00)	—

*CL = contrastive learning

† Comparing against random chance

ǂ Confidence intervals not calculable due to perfect separation

#### Tumor grading performance on histology images enhanced by contrastive learning

To improve upon the baseline histology-based model, we incorporated the histology encoder trained through contrastive learning into the classification framework. With this enhancement, the model achieved a numerically improved AUC of 0.83 (95% CI: 0.60, 1.00) on the test set. Notably, DeLong’s test indicated that the model’s predictions were significantly better than random chance (*p* = 0.002), demonstrating significance above chance.

The enhanced model showed improved accuracy (0.77 vs. 0.69) and numerically higher sensitivity (1.00 vs. 0.33), although specificity was somewhat lower (0.70 vs. 0.80) compared to the baseline model (Table 3). DeLong’s test comparing models before and after contrastive learning enhancement did not indicate a statistically significant change in AUC (*p* = 0.41), although a performance difference was observed. These results suggest that incorporating complementary information from radiographic images via contrastive learning could enable the histology model to better capture clinically meaningful patterns associated with tumor grade, warranting further validation in larger cohorts.

#### Multimodal model performance using both histology and radiographic images

Given that histological examination is typically performed following radiographic imaging, we developed a multimodal model to fully utilize both data sources when available. This integrated model combined (1) pretrained encoders from both histology and radiographic modalities, (2) contrastive learning-enhanced representations, and (3) fusion layers to effectively merge features from both imaging modalities.

When evaluated on the subset of patients with both histology and radiographic data available, the multimodal model achieved an AUC of 1.00 on testing set (AUC = 1.00 on validation sets across the 5 folds) for the chondroid bone tumor grading task, suggesting that combining radiographic and histological features numerically enhances predictive performance. Given the very small sample size (*n* = 11), this result should be interpreted as exploratory and hypothesis-generating rather than evidence of definitive performance superiority, and further validation on larger independent cohorts is essential to confirm the robustness and clinical utility of the multimodal approach in real-world clinical settings.

### Ablation study results

#### Backbone architecture performance for radiograph models

Table 4 summarizes the performance of different backbone architectures for radiograph models across diagnosis and grading tasks. Larger models (InceptionV3, ResNet-50, EfficientNet-B5, B7) were trained with frozen pretrained layers to reduce overfitting, while smaller models (ResNet-18, EfficientNet-B1, B3) had pretrained layers fine-tuned.

**Table 4:** Backbone architecture comparison for radiograph-based models. All values represent AUC with 95% confidence intervals in parentheses

Architecture	Diagnosis AUC (95% CI)	Grading AUC (95% CI)
ResNet-18	0.85 (0.74, 0.97)	0.83 (0.63, 1.00)
ResNet-50	0.87 (0.76, 0.98)	0.83 (0.61, 1.00)
EfficientNet-B1	0.89 (0.80, 0.99)	0.86 (0.65, 1.00)
EfficientNet-B3	0.88 (0.77, 0.99)	0.86 (0.57, 1.00)
EfficientNet-B5	0.86 (0.75, 0.98)	0.83 (0.59, 1.00)
EfficientNet-B7	0.85 (0.73, 0.97)	0.88 (0.71, 1.00)
InceptionV3	0.91 (0.82, 1.00)	0.86 (0.62, 1.00)

InceptionV3 achieved the highest AUC (0.91) for diagnosis, while EfficientNet-B7 performed best for grading (AUC 0.88). InceptionV3 was chosen for subsequent steps due to its superior diagnostic and comparable grading performance, ensuring consistency across models. This choice also aligned with the clinical workflow, as diagnostic models could not be further enhanced with contrastive learning or multimodal fusion, unlike grading models.

Overall, larger frozen architectures outperformed smaller fine-tuned ones, with InceptionV3 consistently showing strong results, supporting its use for later contrastive learning and multimodal fusion.

#### Histology model variants

We evaluated the performance of MaskHIT and Prov-GigaPath under different training strategies. With fine-tuning at a reduced learning rate, MaskHIT outperformed Prov-GigaPath for tumor grading (AUC = 0.73 vs. 0.69), likely due to its smaller architecture generalizing better with limited data. Unfreezing the pretrained encoder and allowing fine-tuning also consistently improved performance compared to keeping the encoder frozen (MaskHIT: AUC = 0.73 vs. 0.62; Prov-GigaPath: AUC = 0.69 vs. 0.67), demonstrating the benefit of task-specific adaptation for histology-based tumor grading.

## Discussion

This pilot study establishes important proof-of-concept for applying contrastive learning to chondroid bone tumor diagnosis and grading, demonstrating that cross-modal knowledge transfer can bring valuable benefits in musculoskeletal oncology. Our methodological contribution shows that contrastive learning holds promising potential to bridge imaging modalities, enabling radiograph-based classification while maintaining flexibility for multimodal enhancement when histological data is available. This represents an encouraging advancement toward more accessible, non-invasive diagnostic strategies as a pilot study. With validation on larger cohorts, such advancement can fundamentally change how we approach chondroid bone tumor assessment in diverse clinical settings. The clinical value of this work is underscored by the inherent challenges in chondroid bone tumor management. These tumors require timely and often aggressive surgical intervention for high-grade lesions, whereas radiographic mimics like fibrous dysplasia and AVN are typically managed conservatively [32, 33]. The diagnostic stakes are particularly high when distinguishing benign enchondromas from malignant chondroid lesions, as this differentiation directly impacts treatment decisions ranging from observation to wide surgical resection [34]. Even multidisciplinary teams using multiple imaging modalities combined with histological evaluation find this distinction challenging, emphasizing the critical need for innovative diagnostic approaches that can provide reliable guidance in complex clinical scenarios.

Our pilot study demonstrates promising performance across three critical clinical applications, each addressing specific gaps in current practice. The radiograph-based diagnostic model achieved promising discrimination (AUC = 0.91) between chondroid bone tumors and their common mimics, suggesting potential utility for initial screening and triage across diverse healthcare settings. More significantly, our contrastive learning approach enhanced radiograph-based tumor grading from AUC 0.86 to 0.95, with particularly notable improvement in sensitivity (1.00 vs. 0.67). Validated in future studies, this enhanced sensitivity can be clinically crucial, as failure to detect high-grade disease could result in delayed treatment and disease progression. When histological data was available, our framework demonstrated consistent trend of improvement through contrastive learning and multimodal integration.

The consistent trend of performance improvements across both imaging modalities provides important mechanistic insights into contrastive learning’s effectiveness for medical imaging applications. Our results suggest that subtle radiographic and histological features can be effectively linked through shared representation learning, enabling knowledge transfer that enhances single-modality performance. The fact that radiograph-based models benefited from histological knowledge during training while requiring only radiographic input at inference demonstrates the practical value of this cross-modal approach. With further validation, this finding has broader implications for medical AI, suggesting that expensive or invasive imaging modalities can be leveraged during training to enhance the performance of more accessible modalities during clinical deployment.

The potential clinical applications of our framework extend across diverse healthcare workflows in musculoskeletal oncology. In primary care or emergency settings, the radiograph-only diagnostic capability could support initial screening and appropriate referral decisions, potentially reducing diagnostic delays and unnecessary referrals. In specialized orthopedic oncology centers, the multimodal approach could enhance existing diagnostic workflows by providing quantitative assessments to complement expert interpretation. The non-invasive nature of radiograph-based assessment is particularly valuable for patient populations where biopsy carries increased risk, for surveillance of multiple lesions, or in resource-limited settings where advanced imaging and pathological expertise may be unavailable.

This pilot study has inherent limitations that define important directions for future research while also highlighting the clinical value of our approach. The relatively small sample size (188 radiographs, 63 histology slides) from a single institution reflects both the rarity of chondroid bone tumors and the challenges of obtaining paired imaging data. In particular, the limited number of high-grade tumors constrained statistical power for grading tasks, resulting in wide confidence intervals for sensitivity estimates. From a clinical applicability perspective, the wide confidence intervals highlight the need for validation on larger, enriched cohorts and prospective study before clinical deployment. At its current stage, the proposed framework should be viewed as a decision-support research tool rather than a standalone diagnostic system. This limitation, however, underscores our approach’s potential value for rare disease applications where large datasets are inherently difficult to obtain. The single-institution design, while limiting immediate generalizability, ensured consistent imaging protocols and expert annotations, providing an optimal controlled environment for establishing proof-of-concept. The promising performance achieved with limited data suggests particular value for rare disease applications where traditional machine learning approaches typically struggle due to data scarcity. Validation across institutions will be essential to confirm generalizability. Future work should integrate clinical metadata, expand to additional tumor subtypes, and prospectively evaluate clinical impact.

Another limitation is the use of radiographic stability over ≥ 2 years as a diagnostic criterion for chondroid tumors, which was adopted to preserve sample size but may allow indolent low-grade chondrosarcomas to be misclassified. Future studies should incorporate longer longitudinal follow-up (e.g., ≥ 5 years) and multi-reader musculoskeletal radiologist consensus to strengthen diagnostic ground truth and reduce potential misclassification.

This work establishes a foundation for future investigations into AI-assisted diagnosis in musculoskeletal oncology, with potential implications beyond chondroid tumors. By showing that contrastive learning achieves promising performance with limited data, it supports democratizing AI diagnosis across diverse settings and rare diseases. The successful transfer of knowledge between imaging modalities suggests broad applicability to other musculoskeletal and medical imaging domains where complementary modalities are used. Ultimately, this work informs future efforts toward accessible, accurate, and timely diagnostic tools that can improve patient outcomes while reducing costs and invasive procedures.

This study establishes important proof-of-concept for contrastive learning in chondroid bone tumor diagnosis and grading, demonstrating that cross-modal knowledge transfer benefits radiograph-based classification while maintaining multimodal flexibility. Our framework achieved encouraging diagnostic performance (AUC = 0.91) and showed a trend toward improved grading accuracy (AUC = 0.95) using radiographs alone, representing a meaningful step forward toward non-invasive tumor assessment. The clinical implications of this pilot study are promising. By enabling classification using only radiographs, our approach may be particularly valuable in resource-limited settings where biopsy or advanced imaging is unavailable or contraindicated, while also allowing integration of histological data when available in diverse clinical workflows. Methodologically, this pilot work demonstrates that contrastive learning can help address key challenges in medical imaging AI for rare diseases, where large datasets are difficult to obtain. The observed success with limited data suggests potential applicability to other musculoskeletal conditions. Future studies should focus on multi-institutional validation to confirm generalizability across different imaging protocols and patient populations. Incorporating clinical metadata, expanding to additional chondroid tumor subtypes, and conducting prospective clinical trials will be essential to assess real-world implementation and impact on clinical decision-making. Overall, this pilot study provides an initial foundation for advancing AI-assisted diagnosis in musculoskeletal oncology, with the long-term goal of supporting more accessible, accurate, and timely diagnostic tools that could improve patient outcomes.

## Supplementary Information

Below is the link to the electronic supplementary material.

Supplementary Material 1

## Data Availability

The datasets generated and analyzed during this study may be made available from the corresponding author upon reasonable request and with appropriate institutional approvals, subject to patient privacy regulations and institutional data sharing policies.

## Abbreviation

AVN: Avascular necrosis
AI: Artificial intelligence
ROIs: Regions of interest
MLP: Multilayer perceptron
AUC: Area under the receiver operating characteristic curve
