# Supplementary material for: Multimodal contrastive learning for non-invasive chondroid bone tumor classification and grading using radiographs
Source: BMC Med Imaging. 2026 Feb 20;26:159. doi: 10.1186/s12880-026-02239-w (PMC13032380; doi:10.1186/s12880-026-02239-w)

**Supplementary Figure 1.** T-SNE plot comparing outcome-based clustering before and after contrastive learning


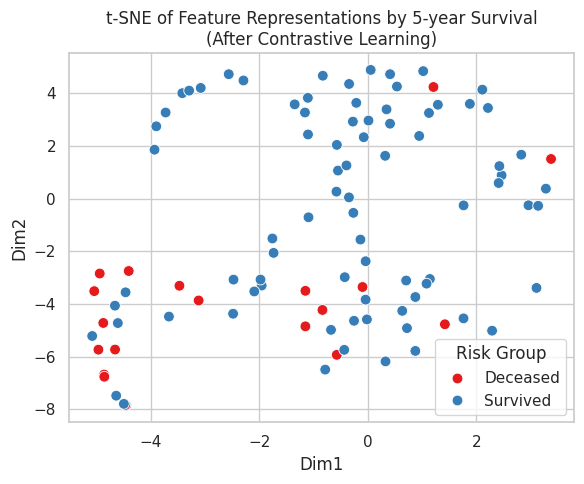

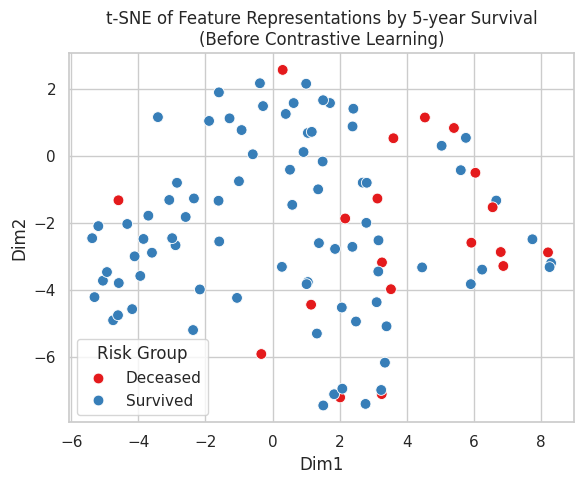

Supplement: Supplementary file 1 — Supplementary Material 1 [file 12880_2026_2239_MOESM1_ESM.docx]
